# Tuberculosis control program in the municipal context: performance evaluation

**DOI:** 10.1590/S1518-8787.2017051006553

**Published:** 2017-03-31

**Authors:** Tiemi Arakawa, Gabriela Tavares Magnabosco, Rubia Laine de Paula Andrade, Maria Eugenia Firmino Brunello, Aline Aparecida Monroe, Antonio Ruffino-Netto, Lucia Marina Scatena, Tereza Cristina Scatena Villa

**Affiliations:** I Programa de Pós-Graduação Enfermagem em Saúde Pública. Escola de Enfermagem de Ribeirão Preto. Universidade de São Paulo. Ribeirão Preto, SP, Brasil; II Departamento Materno-Infantil e Saúde Pública. Escola de Enfermagem de Ribeirão Preto. Universidade de São Paulo. Ribeirão Preto, SP, Brasil; III Departamento de Medicina Social. Faculdade de Medicina de Ribeirão Preto. Universidade de São Paulo. Ribeirão Preto, SP, Brasil; IV Departamento de Medicina Social. Faculdade de Medicina. Universidade Federal do Triângulo Mineiro. Uberaba, MG, Brasil

**Keywords:** Tuberculosis, prevention & control, Program Evaluation, Quality Indicators, Health Care, Evaluation of Health Services

## Abstract

**OBJECTIVE:**

The objective of this study is to evaluate the performance of the Tuberculosis Control Program in municipalities of the State of São Paulo.

**METHODS:**

This is a program evaluation research, with ecological design, which uses three non-hierarchical groups of the municipalities of the State of São Paulo according to their performance in relation to operational indicators. We have selected 195 municipalities with at least five new cases of tuberculosis notified in the Notification System of the State of São Paulo and with 20,000 inhabitants or more in 2010. The multiple correspondence analysis was used to identify the association between the groups of different performances, the epidemiological and demographic characteristics, and the characteristics of the health systems of the municipalities.

**RESULTS:**

The group with the worst performance showed the highest rates of abandonment (average [avg] = 10.4, standard deviation [sd] = 9.4) and the lowest rates of supervision of Directly Observed Treatment (avg = 6.1, sd = 12.9), and it was associated with low incidence of tuberculosis, high tuberculosis and HIV, small population, high coverage of the Family Health Strategy/Program of Community Health Agents, and being located on the countryside. The group with the best performance presented the highest cure rate (avg = 83.7, sd = 10.5) and the highest rate of cases in Directly Observed Treatment (avg = 83.0, sd = 12.7); the group of regular performance showed regular results for outcome (avg cure = 79.8, sd = 13.2; abandonment avg = 9.5, sd = 8.3) and supervision of the Directly Observed Treatment (avg = 42.8, sd = 18.8). Large population, low coverage of the Family Health Strategy/Program of Community Health Agents, high incidence of tuberculosis and AIDS, and being located on the coast and in metropolitan areas were associated with these groups.

**CONCLUSIONS:**

The findings highlight the importance of the Directly Observed Treatment in relation to the outcome for treatment and raise reflections on the structural and managerial capacity of municipalities in the implementation of the Tuberculosis Control Program.

## RESUMO

**OBJETIVO:**

Avaliar o desempenho do Programa de Controle da Tuberculose em municípios paulistas.

**MÉTODOS:**

Pesquisa de avaliação de serviços, com delineamento ecológico, utilizando três agrupamentos não hierárquicos de municípios paulistas de acordo com seu desempenho em relação a indicadores operacionais. Foram selecionadas 195 cidades com no mínimo cinco casos novos de tuberculose notificados no Sistema de Notificação do Estado de São Paulo e 20.000 habitantes ou mais em 2010. A análise de correspondência múltipla foi utilizada para a identificação da associação entre os grupos de distintos desempenhos e as características epidemiológicas, demográficas e de sistemas de saúde dos municípios.

**RESULTADOS:**

O grupo de pior desempenho apresentou as taxas mais elevadas de abandono (média [md] = 10,4; desvio padrão [dp] = 9,4) e as menores proporções de efetivação de Tratamento Diretamente Observado (md = 6,1; dp = 12,9) e esteve associado à baixa incidência de tuberculose, alta confecção tuberculose e HIV, pequeno porte populacional, alta cobertura de Estratégia Saúde da Família/Programa de Agentes Comunitários em Saúde e localização no interior. O grupo de melhor desempenho apresentou a maior taxa de cura (md = 83,7; dp = 10,5) e a maior proporção de casos em Tratamento Diretamente Observado (md = 83,0; dp = 12,7); enquanto o grupo de desempenho regular mostrou resultados regulares de desfecho (cura: md = 79,8; dp = 13,2; abandono: md = 9,5; dp = 8,3) e de efetivação do Tratamento Diretamente Observado (md = 42,8; dp = 18,8). Grande porte populacional, baixa cobertura de Estratégia Saúde da Família/Programa de Agentes Comunitários em Saúde, alta incidência de tuberculose e aids, e localização no litoral e em áreas metropolitanas estiveram associados com esses grupos.

**CONCLUSÕES:**

Os achados destacam a importância do Tratamento Diretamente Observado em relação ao desfecho do tratamento e levantam reflexões sobre a capacidade estrutural e gerencial dos municípios na operacionalização do Programa de Controle da Tuberculose.

## INTRODUCTION

The demand for evidence capable of evaluating the performance of health programs is growing in Brazil^15^. Since the proposed changes in the Health Pact in 2006, the panorama of management policies has strengthened the evaluation of indicators and the negotiation of goals to improve the assistance provided within the context of the Brazilian Unified Health System (SUS)^21^.

However, the evaluation of programs is still a complex task, inserted in a similar complex context of epidemiological reality and the organization of health services present in contemporary society. Certain contagious diseases are closer to the combating logic of chronic diseases than other communicable diseases of rapid course – what characterize them as chronic conditions^14^. Interventions related to the care of chronic conditions include numerous activities involving multiple organizations and professionals over time. In these cases, the difficulty in identifying the responsibility of each one of these elements is greater in relation to the results achieved^23^.

Tuberculosis (TB) is one of the most poignant examples of this situation: an insidious disease, with treatment that lasts at least six months, and which requires, for its activities of control, permanent and coordinated care between different services at various levels of the health system^1^. Despite the *Programa Nacional de Controle da TB* (PNCT – National TB Control Program) formulating and recommending unique guidelines, the implementation of TB Control Programs (PCT) in municipalities is developed in different formats and results because of numerous administrative, political, and geographical specificities of the locations^6^. This peculiarity comes together with the process of decentralization of TB actions to the services of Primary Health Care (PHC), started around the 2000s, which advanced the expansion of the coverage of TB actions in the municipalities. However, at the same time, it generated new challenges for the municipal PCT as it imposes greater permeability in relation to the commitments in the control of the disease and exposes weaknesses in the structure and organization of the entire health system^3^.

There are several possible ways to evaluate the performance on TB control: focus on the performance of specific actions, or on services that operate the program, or on the PCT itself as an agency that gathers certain attributes and activities. Nevertheless, national publications are scarce around performance evaluation of the PCT regarding the program as a whole^9^.

The State of São Paulo is a relevant setting for TB control: it is responsible for approximately 20.0% of the total burden of disease in Brazil and it occupies the sixth place in incidence in the country^a^. In addition, there are several particularities in relation to the organizational context of its health services, including its own system for the notification and monitoring of TB cases, called “TBWEB”^5^. Thus, this study has aimed to evaluate the performance of the PCT in the municipalities of the State of São Paulo.

## METHODS

This is a study of health evaluation with ecological design and descriptive-exploratory approach. We have used the proposed normative evaluation of programs developed by Gonçalves^6^. This proposal suggests the use of a cluster analysis (CA) for the identification of groups of municipalities with different operational performances, followed by further association between these groups with other data characterizing the context, using multiple correspondence analysis (MCA).

We have considered the municipalities of São Paulo with 20,000 or more inhabitants and which had a minimum of five new TB cases, outside the prison system, notified in the TBWEB in 2010. Of the 466 municipalities with new TB cases reported in 2010, 197 municipalities meet the criteria and were included in the study. With the criteria used, the studied municipalities amounted to 95.6% of all new cases of TB in the State of São Paulo in 2010 (n = 14,486).

The State of São Paulo has its own system for computing information about TB since the 1990s. The State has kept its database even after the national implementation of the *Sistema Nacional de Agravos de Notificação Compulsória* (SINAN – National System of Compulsory Notification of Diseases) in 1998^5^. The data collected in the State of São Paulo using the monthly newsletter and notification form for monitoring include mandatory and essential variables consolidated by other units of the country that use the SINAN. Additionally, data are registered on the discovery of the case, name and age of the identified contacts, information about admissions, and number of doses supervised in the Directly Observed Treatment (DOT). The TBWEB uses an online format, in which data can be entered and seen using the Internet throughout the course of TB therapy and patients have a unique record (eliminating possible duplicates). The system offers several features for analysis (cohort, charts, tables) and communication (ability to send messages) using the same interface as the record of the data. Access to TBWEB is restricted to professionals responsible for the epidemiological surveillance in the municipality. They receive the notification records of the care units that perform the diagnosis and treatment of the cases and issue monitoring reports that will be updated by these teams. The notification and record of the data of the patients under treatment is carried out by health services and professionals active in the care and they follow what is advocated by the PNCT throughout the country.

For the characterization of the performance of the PCT, we have used a selection of operational indicators proposed in the Manual of Recommendations of the Tuberculosis Control Program^b^, as well as in prior research of Gonçalves^6^. They are: Proportion of new cases notified by the municipality of residence; Proportion of new cases of pulmonary TB with sputum smear microscopy at the beginning of the treatment; Proportion of HIV testing among new cases; Proportion of HIV testing in progress among new cases; Proportion of new cases diagnosed with bacteriological confirmation; Proportion of indication of DOT and Proportion of supervision of DOT among new cases; Proportion of communicants examined among the communicants identified in new cases; Proportion of abandonment of treatment among new cases; Proportion of cure among new cases; and, Proportion of death among new cases. Variables related to the above information were collected in the TBWEB in January 2013 and the calculation of the indicators was performed for each of the municipalities of study by two researchers independently. The variables were evaluated previously regarding completeness and they presented more than 80.0% of filling in the information system.

Two municipalities were excluded by the impossibility of calculating at least one operational indicator (when the denominator was equal to zero), resulting in 195 municipalities under analysis. After the calculation of the indicators, we carried out a CA applying the hierarchical method, using the squared Euclidean distance as coefficient of similarity. Ward’s method was used as a grouping strategy, followed by the non-hierarchical method. The hierarchical CA identified a cutoff point of three groups of municipalities with different performances, which was used as a strategy for the implementation of the non-hierarchical method. The power of discrimination of the variables for the formation of the groups was verified by analysis of variance, considering the significance level of 5.0%. We excluded the indicators Proportion of new cases notified by the municipality of residence, Proportion of new cases of pulmonary TB with sputum smear microscopy at the beginning of the treatment, Proportion of new cases diagnosed with bacteriological confirmation, and Proportion of death among new cases as they did not show power of discrimination between the different groups (p > 0.005 in the analysis of variance). For the MCA, the municipalities of the study were characterized in relation to demographic aspects (population size and geographic location determined by the region of the Epidemiological Surveillance Group [GVE] of belonging), local health systems (Coverage of the Family Health Strategy [ESF] and the Program of Community Health Agents [PACS]), as well as the epidemiological situation of TB (prioritization of TB control according to technical note from the Ministry of Health, incidence rate of TB and pulmonary TB) and AIDS (incidence of aids and TB/HIV coinfection). The data needed for this characterization were collected in the TBWEB and on the websites of the Brazilian Institute of Geography and Statistics (http://www.cidades.ibge.gov.br/), Department of Informatics of the SUS (DATASUS – http://datasus.saude.gov.br/), and Department of Primary Care/Ministry of Health (http://dab.saude.gov.br/dab/historico_cobertura_sf) in March 2013. The variables of context of the municipalities were included in the MCA after categorization in three ranges (low, average and high) based on the values of the 30 and 70th percentiles. Such characteristics of the municipalities were included in the model of the MCA as active variables, and the groups of municipalities with different performances were considered as additional variables.

This research has been approved by the Research Ethics Committee of the Escola de Enfermagem de Ribeirão Preto, Universidade de São Paulo (Process CAAE 00630812.8.0000.5393/2012).

## RESULTS

**Figure 1:**
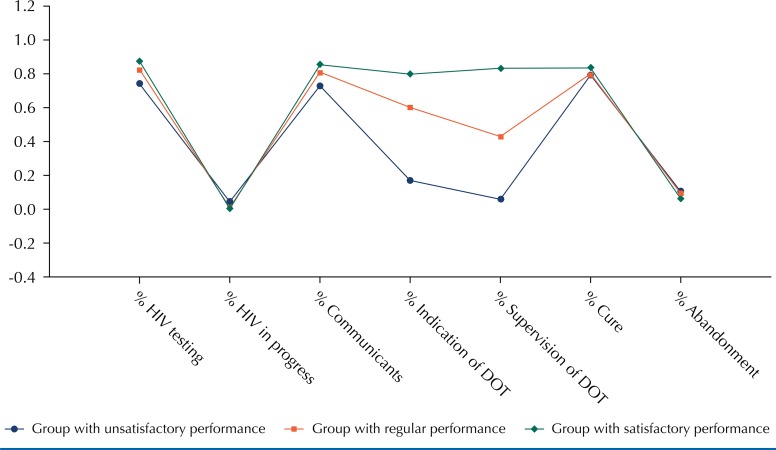
Centroids related to the groups formed by the non-hierarchical method. State of São Paulo, Brazil, 2010.

The group with unsatisfactory performance was composed of 63 municipalities, the group with regular performance had 43 municipalities, and the group with satisfactory performance had 89 municipalities. The characterization of each group was performed by the visualization of centroids (Figure 1) and the averages of the operational indicators for each group (Table). Differences were identified between the three groups (p = 0.0000) in relation to the indication of DOT and its supervision. The groups with unsatisfactory and satisfactory performance showed statistically different averages regarding all indicators studied. In addition to the issues related to DOT, the satisfactory group and the regular group differed in relation to the unsatisfactory group in the proportion of HIV testing in progress (p = 0.0460) (Table).

**Table:** Average operational indicators according to the groups of municipalities. State of São Paulo, Brazil, 2010.

Indicator	Unsatisfactory group (n = 63)		Regular group (n = 43)		Satisfactory group (n = 89)		F-test
		
	Average	(SD)	Average	(SD)	Average	(SD)	
Proportion of HIV testing among new cases	75.0 a	22.6	82.6 ab	13.9	87.3 b	14.6	0.0001*
Proportion of HIV testing in progress among new cases	5.0 a	12.0	1.6 b	3.7	0.6 b	2.3	0.0010*
Proportion of communicants examined among the communicants identified in new cases	73.2 a	25.5	80.7 ab	18.5	85.6 b	17.3	0.0014*
Proportion of indication of Directly Observed Treatment among new cases	17.5 a	15.4	60.5 b	22.0	79.7 c	24.4	0.0000*
Proportion of supervision of Directly Observed Treatment among indicated cases	6.1 a	12.9	42.8 b	18.8	83.0 c	12.7	0.0000*
Proportion of cure among new cases with information on the outcome	78.9 a	10.5	79.8 ab	13.2	83.7 b	10.5	0.0225*
Proportion of abandonment of treatment among new cases with information on the outcome	10.4 a	9.4	9.5 ab	8.3	6.3 b	6.7	0.0056*

Different letters indicate statistically different averages by Tukey test (p < 0.005).

* p < 0.05 by parametric ANOVA test.

**Figure 2:**
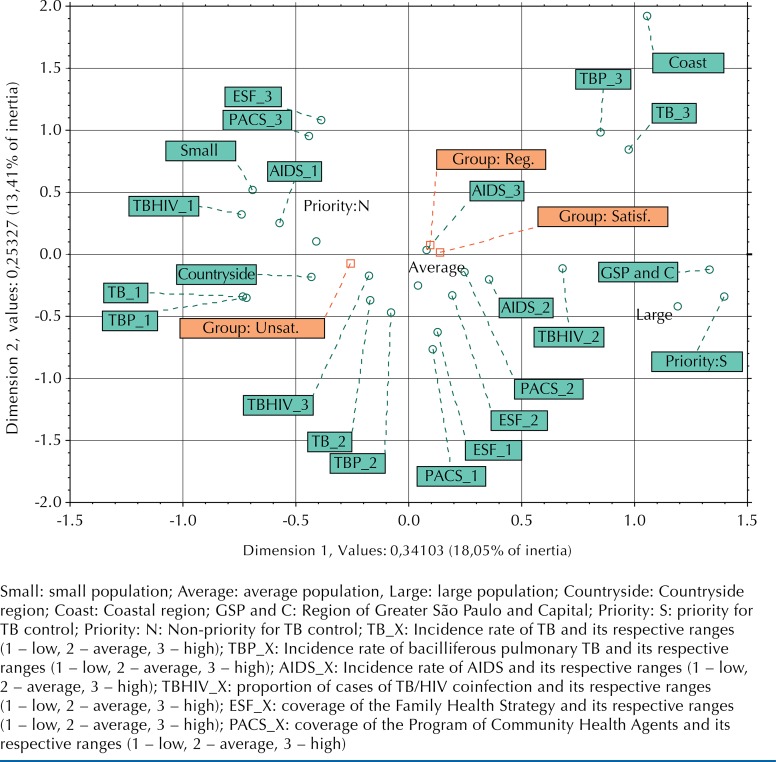
Perceptual map of association between groups, demographic and epidemiological characteristics, and characteristics of the organization of the health system on TB and AIDS, 2010.

In relation to the MCA, we have observed two dimensions, which recovered 33.5% of the total variability of the data (inertia). Dimension 1 consisted of variables that were mostly related to the epidemiology of the disease and to demographic characteristics; dimension 2 consisted of variables related to the characteristics of the health system. The perceptual map (Figure 2) indicates that the poor performance of the PCT (group 1) was associated with the municipalities that present high levels of TB/HIV coinfection, low and average incidence rates of TB and bacilliferous pulmonary TB, being located in the countryside of the State (negative side of dimension 1), as well as with non-priority, small cities, with high coverage of FHS and PACS (positive side of dimension 2). The regular (group 2) and satisfactory performance (group 3) of the PCT was associated with municipalities that had high and average incidence rates of AIDS and moderate TB/HIV coinfection, high incidence of TB and bacilliferous pulmonary TB, being located in the metropolitan region and on the coast (positive side of dimension 1), as well as priority municipalities, with low to average coverage of FHS and PACS (negative side of dimension 2).

Small: small population; Average: average population, Large: large population; Countryside: Countryside region; Coast: Coastal region; GSP and C: Region of Greater São Paulo and Capital; Priority: S: priority for TB control; Priority: N: Non-priority for TB control; TB_X: Incidence rate of TB and its respective ranges (1 – low, 2 – average, 3 – high); TBP_X: Incidence rate of bacilliferous pulmonary TB and its respective ranges (1 – low, 2 – average, 3 – high); AIDS_X: Incidence rate of AIDS and its respective ranges (1 – low, 2 – average, 3 – high); TBHIV_X: proportion of cases of TB/HIV coinfection and its respective ranges (1 – low, 2 – average, 3 – high); ESF_X: coverage of the Family Health Strategy and its respective ranges (1 – low, 2 – average, 3 – high); PACS_X: coverage of the Program of Community Health Agents and its respective ranges (1 – low, 2 – average, 3 – high)

## DISCUSSION

The differences identified in the different performance groups by the CA allow us to reflect on the relationship between the PCT, as a player of the meso-management, and the locus of health services, as micro-management spaces. Among these differences, we highlight the best result of the coverage of the DOT accompanied by higher rates of cure and smaller index of abandonment, which possibly reaffirms the influence of the strategy of the DOT in achieving a favorable outcome for the treatment.

The lower supervision in relation to the total of patients indicated for DOT was expressive in the unsatisfactory and regular groups, indicating a weakness in the development of the strategy. Several issues related to the implementation of the DOT come from the management of municipal coordinators and depend on the articulation between players and sectors. Among them we can mention the offering of incentives, such as food stamps, breakfast, and transportation vouchers, as well as human resources in sufficient number and vehicles for home visits^7^. The availability of these instruments influences the acceptability of health professionals in relation to the practice and the sustainability to carry them out in the daily service and must be considered to understand the results of this study.

Despite the different experiences recorded under the name of the DOT, the strategy goes beyond the simple observation of drug intake. It must be considered as a care management technology with an integrated and humanized approach^16^. Thus, in addition to the organization and availability of the resources mentioned, the good performance in the supervision of the DOT requires a reordering of the care practices and the process of work of health professionals^13^.

In this study, favorable results related to anti-HIV testing among new TB cases reached a proportion above 75.0% even among the group with the worst performance. Such results can be explained by the time of implementation of the anti-HIV rapid test in the network of services in São Paulo – started in mid-2006^19^. The training of the teams responsible for monitoring the TB cases, to carry out the testing in priority municipalities^19^ – which coincide with the areas associated with good performance (coast and Greater São Paulo) –, can signal the benefits of previous experiences of partnerships between the program of AIDS and TB control.

The control of communicants also presented considerable proportions even in the group of unsatisfactory performance. We question the process to produce the data used to calculate the indicator. The under detection of intra-household communicants, the gaps in the clinical evaluation of contacts, and the low number of tests are mentioned in a study carried out in the South of Brazil, which raises the need for more research to understand the results^10^.

Regarding the results identified by the MCA, we need to consider the structural conformation of the network of services, the dynamics of the care model, and the issues that delves into the relations between the meso- and macro-management (level that corresponds to the sphere of health policies and the decision-making and regulatory power).

The results show that the high incidence of TB and AIDS are epidemiological aspects of the prioritization of actions of control, which serve as a formal tool of visibility of the problems surrounding the disease. The association between unsatisfactory performance and high TB/HIV coinfection points to the need for increased vigilance and improvement in the management of cases that are more vulnerable and at greater clinical and abandonment risk even in places with less burden of TB.

Small municipalities generally have lower population qualification in management positions and functions and low human resources, while large municipalities have greater managerial and regulatory capacity^22^. These differences may explain the association of better performance with larger populations, as they more frequently have the instruments to maintain the political commitment around the operationalization of the TB program and ensure the resources necessary for the PCT.

Both in large and in small municipalities, the administrative discontinuity, the lack of autonomy in executive decisions, the political patronage, and the conflict of interests between the different political-party options affect the degree of cooperation achieved between the macro and meso-management. They are problems commonly faced by coordinators of TB programs in the execution of their activities^18^.

Larger municipalities and in urban regions usually have a wider range of own/contracted services. They have a greater trend in the organization and offer of care of medium and high complexity^8^. On the other hand, these municipalities have greater difficulty to implement strategies, such as the FHS and PACS, either because of insufficient population coverage by the PHC, or because of ineffective reference systems between the levels of complexity, or because of the low presence of professionals in these services^4^. The very dynamics of the health-disease process and the use of health services in major urban centers indicate a pattern of health care consumption based on specialization, which weakens the implementation and sustainability of the PHC^8^.

The association between increased coverage of the FHS and PACS and the group of municipalities with the worst performance may suggest that strategies for the strengthening of the PHC do not always produce actions in line with the needs of their territories. In addition, the lack of professional expertise in the management of cases may lead to unfavorable results, a discussion also raised in the article of Yamamura et al.^24^

The consolidation of a new care model in the PHC is not only characterized by the singularities of the horizontal dimension of extension as expressed by the coverage of strategies, such as the FHS and PACS, but also by the vertical dimension of the depth of its institutionalization^14^. We need to consider the discrepancy between the conception of the PHC in the design of the national policy and its expression in local realities^4,11^. In addition, the decentralization of TB actions for the PHC is characterized as a deconcentration of activities in a scenario of high turnover of professionals and accumulation of functions^3,4^. Unsatisfactory performances of TB control are an important message in relation to the quality of the health care and the care models practiced in the reality of health services. That is because the subjects with TB experience the weaknesses of the public health system as a whole in greater frequency and intensity.

Although this study has selected the coverage of the FHS and PACS as a strategic indicator for the characterization of the health system, the decentralization of control actions for PHC cannot be analyzed separately^13^. We need to consider the laboratory support, the flows, the communication between levels, that is, the network needs to be organized around a care line, which enables the coordination of the care provided in all its phases and facilitates the appropriate service for every need/profile of Tb case^2,12^.

In the context of the meso-management, the coordinator of the PCT must know the health care network and how the process of work of teams and services that will detect, diagnose, and treat TB cases is carried out. In addition, the management team of the PCT must articulate the inclusion of the subject of TB in the existing matrix of strategies and guide the disease and its control in permanent education activities^17^. These tools can be a way to face the lack of knowledge on TB by the professionals working on PHC^17^.

The coordinators in TB must strengthen their management action by incorporating instruments such as health plans and reports, valuable documents that give public knowledge on health actions, and by planning actions of indirect (such as routines related to the analysis of the information available) and direct monitoring (such as visits/meetings with health professionals and other players involved in the provision of care)^20,c^. The PNCT itself promotes activities of monitoring and evaluation since 2000, making visits to priority States and municipalities in order to contribute politically and technically to improve the responsiveness to TB. This is a positive example of the partnership between different federated entities whose model can serve as a reference to local managers^b^.

The method of evaluation developed provides possibilities for understanding the performance of local PCT. However, we assume as a weakness the unknown values of other indicators or variables able to explore in greater depth the context in which health interventions are performed. Future studies are needed on this subject.

## Footnotes

a Ministério da Saúde (BR), Secretaria de Vigilância em Saúde, Departamento de Vigilância Epidemiológica. Boletim Epidemiológico. Perspectivas brasileiras para o fim da tuberculose como problema de saúde pública. V.47, n.13, 2016.

b Ministério da Saúde (BR), Secretaria de Vigilância em Saúde, Departamento de Vigilância Epidemiológica. Manual de recomendações para o controle da tuberculose no Brasil. Brasília (DF); 2011.

c Ministério da Saúde (BR), Secretaria de Vigilância em Saúde, Departamento de Vigilância Epidemiológica. Experiências de monitoramento e avaliação no controle da tuberculose no Brasil. Brasília (DF); 2013.
